# Like a Phoenix Reborn from the Ashes: TASK-5 - Commentary to Rinne & Schick et al.

**DOI:** 10.1007/s00424-024-03057-1

**Published:** 2025-01-08

**Authors:** Guiscard Seebohm

**Affiliations:** Institute for Genetics of Heart Diseases (IfGH), Department of Cardiovascular Medicine, University Hospital Münster, 48149 Münster, Germany

The acid-sensitive K_2_P channels, including TASK-5, have been a subject of intense research since its first mentioning in 2001 [2]. Unlike TASK-1 and TASK-3, which contribute to resting membrane potential and are implicated in processes such as anesthesia and ischemia, TASK-5 appeared non-functional when expressed as a homodimer. Rinne & Schick et al. now demonstrate that TASK-5 becomes functionally active only through heterodimerization with TASK-1 or TASK-3. These heteromeric complexes exhibit altered biophysical characteristics, including modified single-channel conductance and responsiveness to Gq-coupled receptor-mediated inhibition. An essential aspect of this study is that the previously known polymorphism G95E in *KCNK15* affects the pharmacology in heteromeric complexes of TASK-1 with TASK-5. This discovery has significant implications for drug development targeting K_2_P channels, emphasizing the need to consider TASK-5's influence on the activity and modulation of TASK-1 and TASK-3. Rinne & Schick et al. used a combination of electrophysiological recordings, biochemical assays, and pharmacological profiling to unravel the functional contributions of TASK-5 and its polymorphism TASK-5^G95E^. The study reveals that heterodimerization with TASK-5 alters the surface expression of TASK channels, regulating their availability and activity. The study also introduces novel pharmacological tools that differentiate between TASK-1/TASK-3 homodimers and TASK-1/TASK-5 heterodimers, providing new tools to studying TASK channels in native and in pathological contexts. The findings have significant biomedical implications, particularly in the context of malignancies linked to KCNK15 polymorphisms. TASK-5-containing heterodimers may influence ion channel activity in tumor microenvironments, offering a novel potential target for therapeutic interventions. Furthermore, the altered pharmacological profile of TASK-1/TASK-5 heterodimers highlights the complexity of developing K_2_P channel modulators, which must account for the heteromeric diversity of these channels. TASK-5’s newly discovered role could provide insights into previously unexplained (neuro-) physiological phenomena attributed solely to TASK-1 and TASK-3. For instance, TASK-5 may contribute to neuronal excitability, pH sensitivity, or anesthetic response in tissues where its expression overlaps with other TASK channels. This work opens several avenues for further research. Firstly, understanding the structural basis of TASK-5 heterodimerization could provide clues for design of novel selective modulators that target specific homomeric or heteromeric TASK complexes. Structural studies using cryo-EM or crystallography may reveal the conformational changes that enable TASK-5 to modulate channel function (Fig. 1). Additionally, exploring the distribution of TASK-5 across tissues could identify its roles in physiological and pathological states. Further, the discovery also raises questions about the regulation of TASK-5 expression and its interaction with broader interactome including other K_2_P channels.

**Fig. 1:**
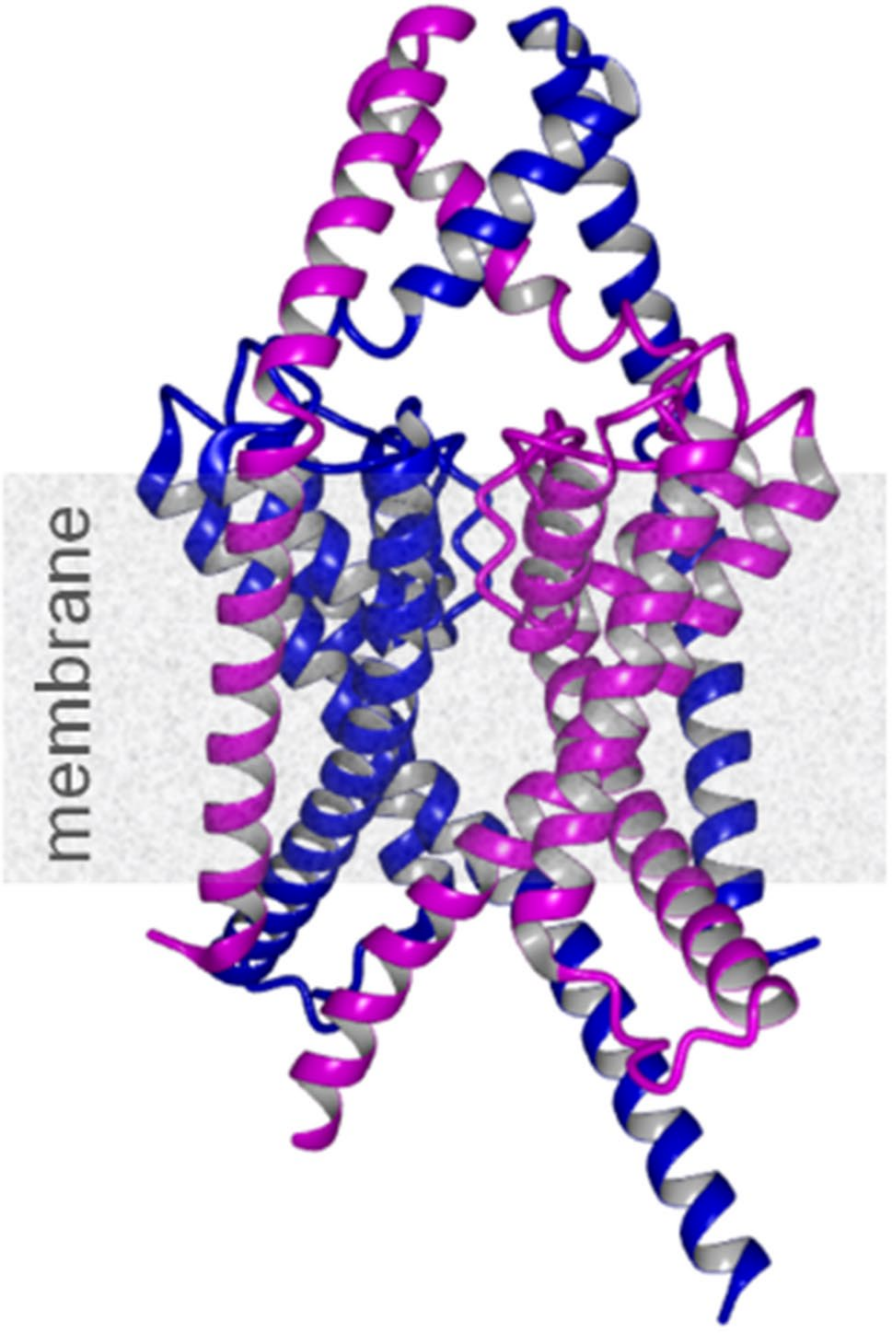
AlphaFold3 model of heteromeric human TASK1/5 complex (coloring: TASK1^1−270^ in blue; TASK5.^1−260^ in magenta)

Rinne et al.'s study fundamentally shifts our understanding of TASK-5, elevating it from an enigmatic, non-functional protein to a crucial modulator of potassium channel activity. The ability of TASK-5 to form functional heterodimers with TASK-1 and TASK-3 underscores the importance of studying ion channels in their native heteromeric configurations. The implications for pharmacology, neuroscience, and oncology are profound, providing new directions for therapeutic development and a deeper understanding of K_2_P channel physiology. This work exemplifies the power of integrative approaches in resolving long-standing biological questions and highlights TASK-5 as a promising target for future research.

## Data Availability

No datasets were generated or analysed during the current study.
